# Silicon as a Sustainable Option to Increase Biomass With Less Water by Inducing Carbon:Nitrogen:Phosphorus Stoichiometric Homeostasis in Sugarcane and Energy Cane

**DOI:** 10.3389/fpls.2022.826512

**Published:** 2022-04-12

**Authors:** Gelza Carliane Marques Teixeira, Renato de Mello Prado, Antonio Márcio Souza Rocha, Marisa de Cássia Piccolo

**Affiliations:** ^1^Laboratory of Plant Nutrition, Department of Agricultural Sciences, São Paulo State University, São Paulo, Brazil; ^2^Laboratory of Biogeochemistry, Department of Technology, São Paulo State University, São Paulo, Brazil; ^3^Laboratory of Nutrient Cycling, Center of Nuclear Energy in Agriculture, University of São Paulo, São Paulo, Brazil

**Keywords:** beneficial element, abiotic stress, nutritional homeostasis, *Saccharum officinarum* L., *Saccharum spontaneum* L.

## Abstract

Climate change has prolonged periods of water deficit in sugarcane and energy cane crops. This condition induces an imbalance of the carbon (C): nitrogen (N): phosphorus (P) stoichiometric homeostasis, impairing accumulated nutrients from being converted into biomass. Silicon (Si) supplementation can mitigate the damage caused by water deficit in plants by improving the C:N:P balance, increasing C, N, and P use efficiencies and the biomass conversion, and reducing climate change effects on crops. This study assesses the beneficial effects of Si applied through fertigation associated with foliar spraying on the alleviation of damage caused by severe water deficit in sugarcane and energy cane for intermediate and long periods. In addition, the effects in maintenance of nutritional homeostasis we assessed and C, N, and P use efficiencies on sugarcane and energy cane under those conditions were increased. Four experiments were conducted during the first growth cycle of each species. The effect of fertigation associated with Si foliar spraying was evaluated by applying Si only during the seedling formation phase in sugarcane and energy cane grown under severe water deficit for 60 days after transplanting (intermediate period). Then, the effect of Si applied during seedling formation and supplemented after transplanting was evaluated in sugarcane and energy cane grown under severe water deficit for 160 days after transplanting (long period). The Si supply decreased C contents, modified the C:N:P ratio, and increased C, N, and P use efficiencies in plants of both species under water deficit at the intermediate and long periods after transplanting. The effects of applying Si through fertigation associated with foliar spraying during seedling formation mitigated the damage caused by severe water deficit in the intermediate period, which was mainly observed in sugarcane. When supplemented with Si after transplanting, the mitigating effects occurred in both species under severe long period water deficit. Therefore, the Si supply through fertigation associated with foliar spraying is a viable alternative to provide Si to the plant. It also comes with beneficial effects that partially reverse the damage to nutritional homeostasis and increase nutritional efficiency in plants under severe water deficit.

## Introduction

Sugarcane (*Saccharum officinarum* L.) is used in sugar and alcohol production and can accumulate high sucrose concentrations (Ferreira et al., 2017). The sugar-energy industry has been expanding the second-generation bioelectricity and bioethanol production by introducing new species such as energy cane (*S. spontaneum* L.) which has high total fiber content, low sucrose content, and high dry matter production per area (Cursi et al., 2021). The areas aimed at sugarcane and energy cane crops have been expanding to regions with lower water availability and are expected to increase in the future due to climate change (Besharat et al., 2020). Water deficit is more intense in plants from regions with sandy soils and those with low water retention capacity (Schaller et al., 2020), such as Entisols (Quartzipsamments) and especially in main producing countries such as Brazil (ANA, 2021), China (He et al., 2021), and India (Dingre et al., 2021).

The water deficit promotes imbalance in water status (Teixeira et al., 2020a) and decreases photosynthetic rate (Ahmad et al., 2021; Verma et al., 2021), causing severe damage to the plant. At the molecular level, water deficit increases reactive oxygen species (ROS) such as superoxide ion (O_2_^–^), hydroxyl radical (HO), and hydrogen peroxide (H_2_O_2_) (Yin et al., 2005). Chlorophyll degradation and membrane lipid peroxidation are associated with increased ROS production (Ahmad et al., 2021). ROS also affects biochemical processes that interfere with the action of enzymatic (Bezerra et al., 2019) and non-enzymatic defense antioxidants (Teixeira et al., 2021) such as glutathione, carotenoids, proline, and ascorbic acid (Mahmood-Ur-Rehman et al., 2020). Consequently, plant growth and dry matter production are also affected. Furthermore, Silicon (Si) can also modulate chlorophyll metabolism and redox homeostasis and regulate nutrient uptake in mustard plants, leading to the delay of premature leaf senescence under salinity and water deficit (Alamri et al., 2020).

New approaches to the effects of water deficit on plants have emerged regarding the Carbon (C): Nitrogen (N): Phosphorous (P) stoichiometric modification. The study of stoichiometry involves evaluating chemical element homeostasis, especially C, N, and P in plant systems (Elser and Hamilton, 2007). Water deficit can reduce the ion-root contact and the nutrient movement in the soil and, consequently, its uptake and metabolism in plants by decreasing their performance of biological functions and conversion rate into dry matter (Ma et al., 2020). Therefore, water deficit leads to nutritional imbalance by modifying the elemental stoichiometric homeostasis and decreasing the nutrient use efficiency in the plant, especially nutrients with structural functions, such as C, N, and P. Those elements make up many essential organic compounds due to having great influence on proper metabolism and, consequently, on optimal plant growth (Brouder and Volenec, 2008). This influence was responsible for the decrease in the growth of very young sugarcane (Teixeira et al., 2020b). Although longer dry periods are common in sugarcane (Cardozo et al., 2018) may affect plants in more advanced stages of development, with a probable worsening of this nutritional imbalance.

The use of Si is an option to reduce the damage caused by water deficit in sugarcane. However, studies only predominantly evaluate the physiological processes of plants (Bezerra et al., 2019; Teixeira et al., 2020a,^2021^; Camargo et al., 2021; Verma et al., 2021). Similar to other Poaceae plants such as rice, sugarcane can also actively uptake Si (Mitani-Ueno and Ma, 2021). The Si present in plants is prevalent in cell walls as amorphous silica (Neethirajan et al., 2009) and in organic complexes with cellulose (Tombeur et al., 2020), hemicellulose (He et al., 2015), and other cell wall components, suggesting that Si plays an important role in increasing plant growth (Isa et al., 2010). The formation of these Si-organic compounds in the cell wall is a synthesis with low energy cost (Hao et al., 2020), and unlike lignin, which has a high energy cost, this has synthesis can be decreased by Si signaling (Schoelynck et al., 2010). Furthermore, it induces the C content to be reduced in the cell wall and may reflect in the leaves of plants that received Si by modifying the stoichiometric C:Si ratio, especially under water deficit found in forage plants (Rocha et al., 2021). This phenomenon can also occur in non-stressed plants such as wheat crops (Neu et al., 2017). Therefore, future research must focus on the specifics of Si and plant interactions for exploitation and improvement of crop production (Ahanger et al., 2020).

The beneficial effect of Si in alleviating water deficit in sugarcane plants would be due to the elemental stoichiometric homeostasis involving, in addition to C, the structural nutrients N and P, favoring the balance of the nutritional functions and nutritional efficiency of these elements in plants. Nevertheless, there is still much to be investigated about the Si effects on this Poaceae to alleviate the water deficit damage, as there is only one study in young sugarcane plants that was developed in a very limited 30-day period of water stress (Teixeira et al., 2020b). Thus, there is doubt whether a longer period of water deficit of 2 or even 5 months, which is more common in crops growing sugarcane or energy cane, could nullify or decrease the benefit of Si on the C:N:P stoichiometric homeostasis and the gain of plant biomass. To prolong the effect of Si, the efficiency of application and element uptake by the plant needs to be increased, either during the formation of pre-sprouted seedlings or after its transplanting to the soil.

The seedling is produced from a mini stem, particularly a small fraction of the stem containing a bud used to originate a pre-sprouted seedling. This is an important innovation in the sugarcane propagation system. The seedling is grown in an inert substrate in a nursery for 60 to 70 days after the emergence of sprouts. The stage after transplantation is very sensitive, with a high risk of seedling death due to low water reserves. Hence, Si supply can promote large enrichment of Si in the seedlings. This would increase a possible beneficial residual effect of Si to alleviate severe water deficit damage for up to 60 days after transplantation (Dingre et al., 2021). It is noteworthy that about 1/3 of the plant leaves are cut at this stage before transplanting to the soil to reduce water loss (Landell et al., 2012), decreasing the amount of beneficial elements in the seedling. This study also evaluates whether fertigation and foliar spraying of Si in the seedling production phase and supplementary applications after transplanting into the soil can prolong the effects induced by Si in alleviating the damage caused by severe water deficit until the initial stem elongation phase during the relatively long period of up to 160 days after transplantation. This water deficit period is critical as it coincides with the crop’s high relative growth rate (Dingre et al., 2021) and can affect the next phenological phases, mainly in the complete elongation of the stem of the species studied.

Given the lack of information on sugarcane and energy cane, we hypothesized that the water deficit damage to dry matter production is due to an imbalance in nutritional homeostasis, implying decreased C, N, and P use efficiencies. However, the Si supply may reverse such an imbalance and have its residual beneficial effect prolonged, especially when the plant receives more Si in complementary applications after transplanting than in seedling production phase only, but this can also depend on the species. Corroborating this hypothesis will help to better understand the mechanisms of Si in alleviating the water deficit for a relatively long period after transplantation (up to 5 months) and to identify whether there is a species that benefits more from Si. Thus, this research can point to Si as a new strategy for managers in the sugar-energy sector to promote policies aimed at greater biomass production and more water sustainability.

Thus, this study evaluates the beneficial effects of Si applied through fertigation associated with foliar spraying on the alleviation of severe water deficit damage at 60 and 160 days after transplanting on the maintenance of nutritional homeostasis and on the increase of C, N, and P use efficiency in sugarcane and energy cane.

## Materials and Methods

### Plant Material and Growing Conditions

Four experiments were simultaneously developed in a greenhouse at the Universidade Estadual Paulista (UNESP) Jaboticabal campus from January to September 2019 using plants of two species: *Saccharum officinarum* L. (sugarcane - variety RB 966928 - experiments I and III) and *S. spontaneum* L. (energy cane - variety VX2 - experiments II and IV). The energy cane variety that was used was obtained from Vignis^®^ and is classified as type II for producing high fiber (>28%) and low sugar content in the form of sucrose (<6%) (Matsuoka et al., 2014).

The experiments were developed by initially growing both species’ sprouts (up to 70 days after the complete emergence) in pots filled with fine vermiculite that received the Si treatments during the seedling formation phase. Then, they were transplanted into pots with Entisols (Quartzipsamments) samples and received the treatments with the water regimes.

### Treatments and Experimental Design

The treatments were arranged in a 2 × 2 factorial scheme consisting of two forms of Si supply: absence (-Si) and presence of Si (+Si) applied via fertigation associated with foliar spraying. Furthermore, there were two soil water regimes: 70% of the soil water retention capacity (WRC) (control - without deficit) and 30% of the WRC (severe water deficit, WD).

Experiments I and II used sugarcane and energy cane, respectively, with Si applied exclusively in the seedling production phase and WD applied from 7 to 60 days after transplanting (intermediate period). Experiments III and IV used sugarcane and energy cane, respectively, with Si applied in the seedling production phase and supplemented after transplanting. WD was applied from 7 to 160 days after transplanting (long period). The plots in all experiments were arranged in randomized blocks, with 6 repetitions in experiments I and II and 5 repetitions in experiments III and IV.

Pre-sprouted seedlings of both species that were studied were initially produced for treatment validation. The mini stems (5 ± 0.5 cm long) were planted in seedling production trays filled with fine vermiculite. Nutrient solution, according to a Hoagland and Arnon (1950), was used with a change in iron concentration of up to 368 μmol L (Cavalcante et al., 2016) in the Ethylenediamine di-2-hydroxyphenyl acetate ferric (Fe-EDDHA) form. In order to avoid the substrate’s salinization, the nutrient solution concentration during the first week of cultivation was kept at 25% dilution and increased to 50% from the second week until the end of the seedling formation phase. In order to determine the volume of the solution to be applied during this stage, a saturation test was previously performed, and the volume of 10 ml per cell was sufficient to saturate the substrate, avoiding loss by leaching. In addition, the solution’s pH value was adjusted to 5.5 ± 0.2 using a hydrochloric acid solution or sodium hydroxide, both at 1.0 mol L^–1^.

The soluble Si source used was sodium-potassium silicate that was stabilized with sorbitol (113.4 g L^–1^ Si, 18.9 g L^–1^ K_2_O, 60.5 g L^–1^ of Na_2_O, and 100 ml L^–1^ sorbitol, and pH 11.8) at a concentration of 2.5 mmol L^–1^. This Si concentration does not induce polymerization because this process only starts at concentrations higher than 3.0 mmol L^–1^ (Birchall, 1995). Fifteen Si-fertigation were performed at 4-day intervals, starting at 10 days after the complete emergence of sprouts (days after emergence, DAE), applying 10 ml per seedling through the substrate (to induce root uptake), and 1.5 ml per seedling through foliar spraying (to induce foliar uptake). The volume applied per seedling in foliar spraying was obtained by preliminary water testing, taking the consumed volume’s average (5 repetitions) to apply enough fine droplets to cover the entire leaf surface without a runoff. The pH of the solution used in fertigation and foliar spraying was adjusted to 5.5 ± 0.2. The amount of potassium present in the Si source was balanced in the treatments without the element using a 1.0 mol L^–1^ potassium chloride solution applied through the roots and foliar spraying.

At 70 DAE, the second phase of the experiments began. For this purpose, seedlings were transplanted into 5.5 dm^3^ (experiments I and II) and 20.0 dm^3^ (experiments III and IV) pots filled with samples of Entisols (Quartzipsamments) (Soil Survey Staff, 2014) collected from the Ap horizon. According to the method described by Raij et al. (2001), the soil’s chemical analysis was performed for fertility purposes, presenting the following results: pH (CaCl_2_), 4.3; organic matter, 9.0 g dm^–3^; P (ion exchange resin extractor), 2.0 mg dm^–3^; Boron (B), <0.12 mg dm^–3^, Copper (Cu), 0.2 mg dm^–3^; Iron (Fe), 9.0 mg dm^–3^, Manganese (Mn), 1.7 mg dm^–3^; Zinc (Zn), 0.4 mg dm^–3^; Calcium (Ca), 3.0 mmol_*c*_ dm^–3^; Magnesium (Mg), 1.0 mmol_*c*_ dm^–3^; Potassium (K), 0.3 mmol_*c*_ dm^–3^; potential acidity (H + Al), 16.0 mmol_*c*_ dm^–3^; sum of bases (Ca + Mg + K) (SB), 4.0 mmol_*c*_ dm^–3^; cation exchange capacity (CEC), 20 mmol_*c*_ dm^–3^; and base saturation (V: SBx100/CEC), 21.0%. The Si content was 1.0 mg dm^–3^, which was determined using calcium chloride extractor at.01 mol L^–1^ according to the method described by Korndörfer et al. (2004).

Thirty days before transplanting, limestone was applied [total neutralization power: 125%, Ca oxide (CaO): 48%, Mg Oxide (MgO): 16%] in the soil, adequately mixed with the soil volume, to increase the V to 60%. The soil was kept at 70% of WRC to induce the limestone reaction. After this period, fertilizer was applied to the soil using 150 mg dm^–3^ of N, P, and K as ammonium sulfate, triple superphosphate, and potassium chloride, respectively. Triple superphosphate was applied as a single dose and incorporated into the soil volume. Ammonium sulfate and potassium chloride were applied through fertigation in three doses of 50 mg dm^–3^ at 7-day intervals. Also, 5 mg dm^–3^ of Zn as zinc sulfate and 2 mg dm^–3^ of B as boric acid were applied through fertigation in a single dose at the first application of N and K fertilizers.

At transplantation, the seedlings had six fully developed leaves, and a cut was made 30 cm from the sheath of the first fully developed leaf; that is, about one-third of the leaves were removed. This is a common practice in seedling nurseries aimed at decreasing water vapor loss by transpiration at the time of transplanting to the soil. Then, the seedlings were transplanted into pots filled with soil and kept at 70% WRC for seven days and, after that, were subjected to the soil water regimes.

In experiments III and IV, the same Si concentrations and application times described above were performed during seedling formation stage in addition to 5 more Si fertigations and foliar sprayings that were applied after transplanting at 20, 35, 50, 65, and 80 days. The Si application through fertigation was made to simulate a 5 mm blade of silicate solution, with approximately as 500 ml of solution applied per 20 dm^3^ pot, considering a surface area of 962 cm^2^. The concentration of Si used was 2.5 mmol L^–1^ in an approximate volume of 500 ml, which corresponds to approximately 35 mg of Si by pot and is equivalent to 3.6 kg ha^–1^ of Si per application or 18 kg ha^–1^ of Si in the five applications. Foliar spraying was simultaneously performed with fertigation using a handheld sprayer to ensure leaf coverage without a runoff. The fertigation solution pH was adjusted to 5.5 ± 0.2. The amount of potassium present in the Si source was balanced in the treatments using a 1.0 mol L^–1^ potassium chloride solution applied through the fertigation and foliar spraying.

The soil water regimes were determined from the microporosity values obtained by the tension table method with a 60 cm water column. In order to do this, undisturbed soil samples were collected using a volumetric ring (v: 98.125 cm^3^). The samples were saturated for 24 h and then placed on the tension table for 72 h before being weighed (a). Afterward, the samples were dried in a greenhouse at 110°C for 24 h, and the mass was weighed again (b). The total microporosity (Mi): [(a-b)/v] found was equivalent to 100% of the WRC (Embrapa, 1997). However, the optimal water condition was 70% of this value as it allows 70% of the micropores to be filled with available water and the remaining 30% with air, maintaining root gas exchange (Boaretto et al., 2014). Thus, the soil water regimes were: 70% WRC as a control (without deficit) and 30% WRC as a severe WD condition for sugarcane, as indicated by Teixeira et al. (2020a).

Irrigation management was performed daily so that soil moisture within each treatment had the least possible variation, i.e., insignificant to alter the plant’s biological response. Therefore, the soil mass used to fill each pot was strictly controlled. In addition, the pots’ mass was also set to deduct from the total mass. After that, the mass replacement method was used, in which water losses by soil evaporation and plant transpiration were considered and controlled daily by weighing the pots. This ensured that the plants were kept at the proposed WRC levels in the treatments because the adjustments were made in all pots. The same irrigation frequency was used for both species and irrigation treatments (i.e., all were weighed daily).

When the water was replenished, the 70% WRC pots had approximately 60% water, and the 30% WRC pots had approximately 25% water. The higher water loss in the 70% WRC pots is justified by the higher transpiration rate per leaf area caused by the higher plant growth. The irrigations were performed at the same time of day (5 pm) due to preliminary tests that were carried out, and because water loss was more accentuated between the period of 2–5 p.m. Therefore, although there was variation, it only occurred during a period of 3 h a day.

The plants in experiments I and II were grown under severe WD for a period of 7 to 60 days after transplanting (tillering phase) (intermediate period), while those in experiments III and IV were grown up to 160 days (stem’s initial formation phase) (long period). After this period, the plants were collected, and the analyses were performed as described below.

### Analyses Performed

#### Leaf Water Potential (Ψw)

Leaf water potential (Ψw) was determined in the middle third of the blade of the second fully developed leaf with a Scholander pressure chamber (3000F01, Soil Moisture Equipment, United States) at 5 am. The pressure was applied until exudation occurred at the cut site made on the leaf petiole (Turner, 1981).

#### Relative Water Content

Ten discs (26.4 mm^2^) were collected from the first fully developed leaf at 6 am. The discs were immediately weighed to determine the fresh mass (FM). Then, the samples were rehydrated in deionized water for 6 h to obtain turgid mass (TM) and dried in a forced-air circulation oven (TE-394/3-MP, Tecnal, Brazil) at 80°C for 24 h to determine the dry mass (DM). The values were determined by the equation proposed by Barrs and Weatherley (1962): [(FM-DM)/(TM-DM)] × 100.

#### Dry Matter Production

The plants were separated into leaves and stems, washed in drinking water, detergent solution (0.1% v/v), HCl solution (0.3% v/v), and deionized water. The plant material was dried in a forced-air circulation oven (TE-394/3-MP, Tecnal, Brazil) (65 ± 5°C) until it reached a constant mass, and the dry matter of each plant part was obtained.

#### Silicon Content

Silicon content in leaves and stems were obtained by extracting the element using hydrogen peroxide (H_2_O_2_) and sodium hydroxide (NaOH). The reaction was induced in a forced air circulation oven (TE-394/3-MP, Tecnal, Brazil) at 95°C in accordance to the methodology described by Kraska and Breitenbeck (2010). The reading was performed by the colorimetric method with hydrochloric acid, oxalic acid, and ammonium molybdate using a spectrophotometer (B442, Micronal, Brazil) at 410 nm, as indicated by Korndörfer et al. (2004).

#### Carbon, Nitrogen, and Phosphorus Contents

Carbon and N content in leaves and stems were determined by dry combustion at 1,000°C using a LECO Truspec CHNS elemental analyzer, calibrated with a LECO (502-278, Setor Joseph, MI, United States) wheat standard (*C* = 45% and *N* = 2.68%). P content in leaves and stems were determined by sample digestion using a mixture of perchloric acid and nitric acid (1:2), with readings taken by the molybdenum antimony colorimetric method in a spectrophotometer (B442, Micronal, Brazil) at 420 nm (Bataglia et al., 1983).

#### Stoichiometric Ratios Silicon: Carbon:Nitrogen:Phosphorus

The stoichiometric ratios C:Si, C:N, C:P, and N:P were determined from the ratio between the contents of these elements in plant tissues such as leaves and stems.

#### Carbon, Nitrogen, and Phosphorus Use Efficiencies

The C, N, and P use efficiencies that were calculated according to Siddiqi and Glass (1981), which were expressed as the quotient of the squared dry matter by the respective nutrient accumulation. Thus, the following equation was used:


$$\mathrm{Use}\mathrm{efficiency}\left(g^{2}g^{-1}\right)=\left(\mathrm{whole}\mathrm{plant}\mathrm{dry}{\mathrm{matter}}^{2}\right)÷\mathrm{total}\mathrm{nutrient}\mathrm{accumulation}\left(g\mathrm{per}\mathrm{plant}\right)$$

### Statistical Analysis

The experiments were analyzed independently. Data were subjected to two-way analysis of variance (ANOVA) by *F*-test (*p* < 0.05) after meeting the normality assumptions (Shapiro-Wilks *W*-test) and homogeneity of variances (Bartlett’s test).

Factor analysis was used to test the main effects of Si supply, WD, and their interactions (Si × WD). The Tukey test compared the means at 5% probability using the SAS^®^ statistical software (Cary, NC, United States).

## Results

### Silicon, Carbon, Nitrogen, and Phosphorus Content in Leaves and Stems

**FIGURE 1:**
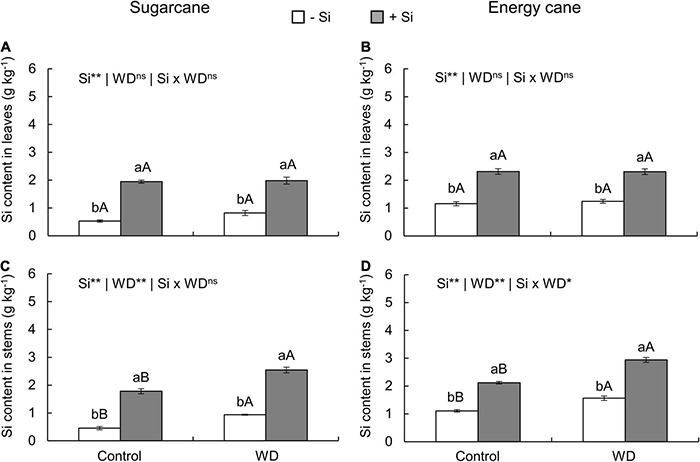
Silicon (Si) contents in the leaves and stems of sugarcane **(A,C)** and energy cane **(B,D)** plants in the absence of Si application (-Si), and with Si applied via fertigation associated with foliar spraying (+Si) under adequate water regime (control) and in water deficit (WD) for a period of 7 to 60 days after transplanting. ** and * significant with 1 and 5% probability, respectively, and ns, not significant by the *F*-test. Lowercase letters indicate differences in relation to Si and capital letters in relation to water deficit. *n* = 6. Si × WD: interaction.

**FIGURE 2:**
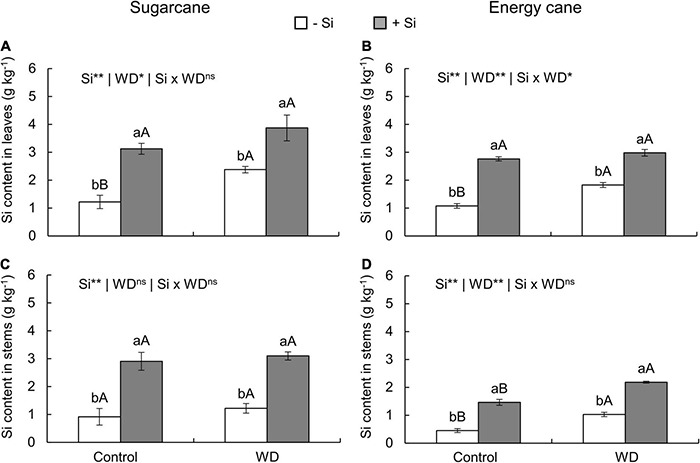
Silicon contents in the leaves and stems of sugarcane **(A,C)** and energy cane **(B,D)** plants in -Si, and with + Si under adequate water regime (control) and in WD for a period of 7 to 160 days after transplanting. ** and * significant with 1 and 5% probability, respectively, and ns, not significant by the *F*-test. Lowercase letters indicate differences in relation to Si and capital letters in relation to water deficit. *n* = 5. Si × WD: interaction.

The Si supply in all experiments, compared to those -Si, promoted an increase in the Si content of leaves and stems (Figures 1A–D, 2A–D).

In -Si, the WD until the tillering phase increased the Si content in sugarcane (experiment I) and energy cane (experiment II) stems compared to control plants (Figures 1C,D). However, with WD extending to the stem’s initial formation phase, higher leaf Si contents were observed in sugarcane (experiment III) and energy cane (experiment IV) (Figures 2A,B), and also in energy cane stems (Figure 2D), compared to control plants.

**FIGURE 3:**
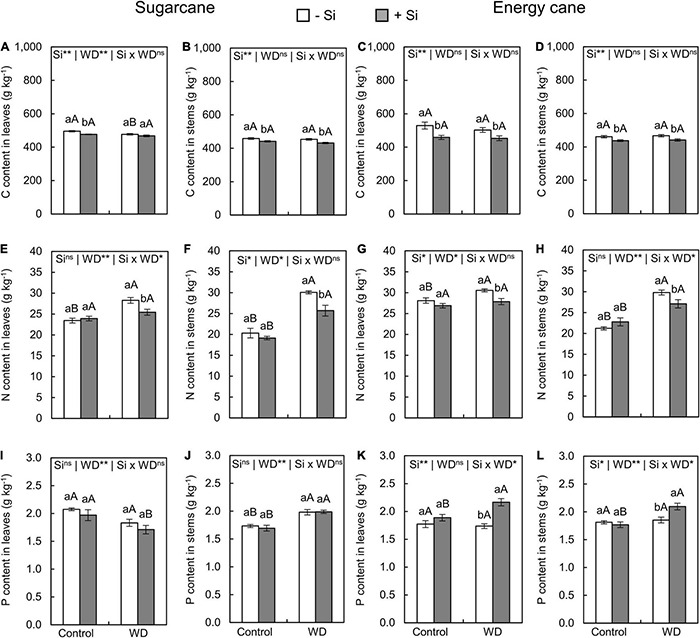
Contents of carbon (C), nitrogen (N), and phosphorus (P) in the leaves and stems of sugarcane **(A,B,E,F,I,J)** and energy cane **(C,D,G,H,K,L)** in -Si, and + Si under adequate water regime (control) and in WD for a period of 7 to 60 days after transplanting. ** and * significant with 1 and 5% probability, respectively, and ns, not significant by the *F*-test. Lowercase letters indicate differences in relation to Si and capital letters in relation to water deficit. *n* = 6. Si × WD: interaction.

**FIGURE 4:**
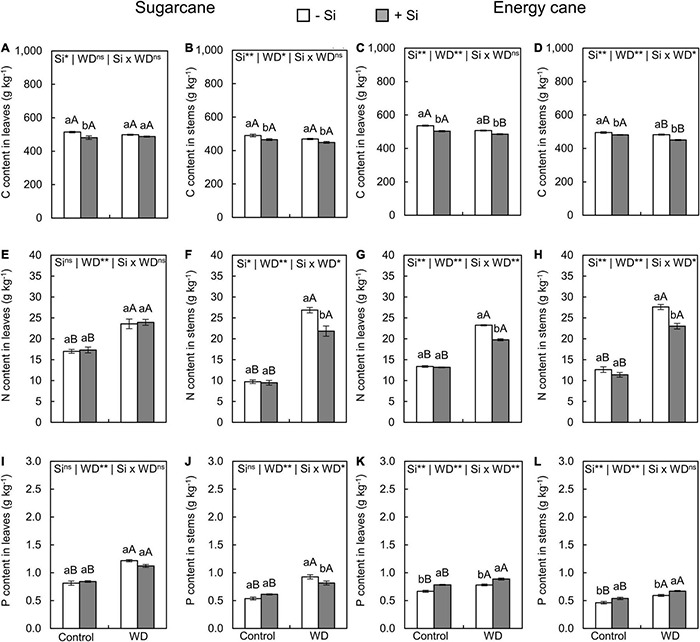
Contents of C, N, and P in the leaves and stems of sugarcane **(A,B,E,F,I,J)** and energy cane **(C,D,G,H,K,L)** in -Si and + Si under adequate water regime (control) and in WD for a period of 7 to 160 days after transplantation. ** and * significant with 1 and 5% probability, respectively, and ns, not significant by the *F*-test. Lowercase letters indicate differences in relation to Si and capital letters in relation to water deficit. *n* = 5. Si × WD: interaction.

Growing plants under -Si and WD until the tillering phase had a decreased C content only in sugarcane leaves (experiment I) compared to control plants (Figures 3A–D). However, the WD until the stem’s initial formation phase only decreased C content in leaves and stems of energy cane (experiment IV) compared with the control plants (Figures 4A–D).

The Si supply in the seedling production phase only and Si supply after transplanting decreased the C content of leaves and stems in sugarcane (experiment I and III) and energy cane (experiment II and IV) grown under the control (Figures 3A–D, 4A–D). Such a decrease in C content of sugarcane stems (Figure 4B) and energy cane leaves and stems (Figures 4C,D) was also found in plants under WD and + Si.

Nitrogen content in leaves and stems increased in plants of both species grown under + Si and -Si in the two periods of WD compared to control plants (Figures 3E–H, 4E–H). However, the N content was lower in leaves and stems of both species under WD and with Si supply (Figures 3E–H, 4E,F), with the exception of the leaves of sugarcane under WD until the initial stem formation phase (Figure 4E).

The exposure of plants to WD until the tillering phase in -Si only increased the P content in the sugarcane stems (Figures 3I–L) compared to the control plants. However, leaves and stems of sugarcane and energy cane had higher P contents under WD until the stem’s initial formation regardless of Si supply (Figures 4I–L).

The Si supply in the seedling formation phase and after the transplanting increased P content in leaves and stems of energy cane (experiment II and IV) under the two water regimes compared to -Si (Figures 4K,L). On the other hand, the P content in stems of sugarcane had decreased under WD until the stem’s initial formation (experiment III) (Figure 4J).

### Stoichiometric Ratios Silicon:Carbon:Nitrogen:Phosphorus in Leaves and Stems

**FIGURE 5:**
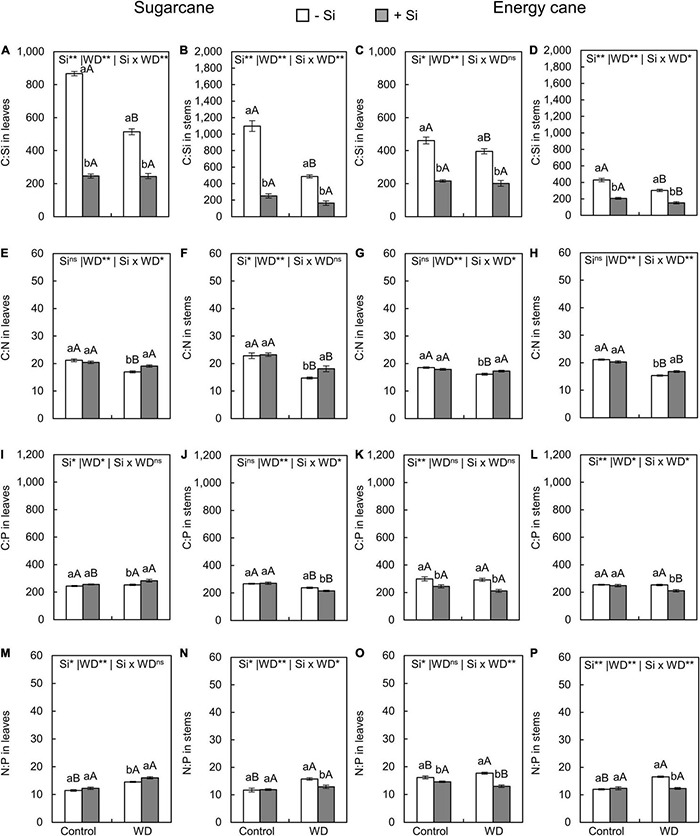
C:Si, C:N, C:P, N:P ratios in the leaves and stems of sugarcane **(A,B,E,F,I,J,M,N)** and energy cane **(C,D,G,H,K,L,O,P)** in -Si and + Si under adequate water regime (control) and in WD for a period of 7 to 60 days after transplantation. ** and * significant with 1 and 5% probability, respectively, and ns, not significant by the *F*-test. Lowercase letters indicate differences in relation to Si and capital letters in relation to water deficit. *n* = 6. Si × WD: interaction.

**FIGURE 6:**
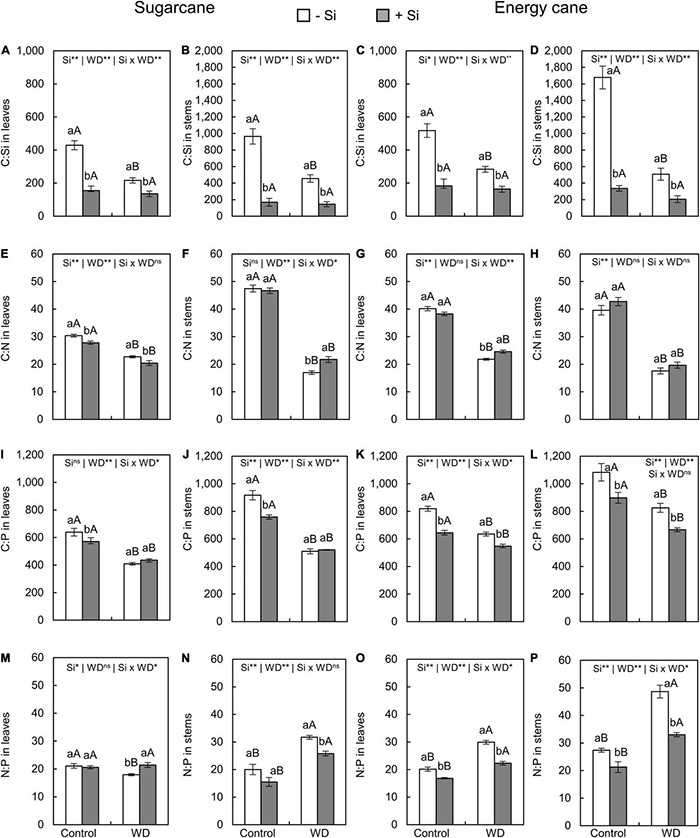
C:Si, C:N, C:P, N:P ratios in the leaves and stems of sugarcane **(A,B,E,F,I,J,M,N)** and energy cane **(C,D,G,H,K,L,O,P)** in -Si and + Si under adequate water regime (control) and in WD for a period of 7 to 160 days after transplantation. ** and * significant with 1 and 5% probability, respectively, and ns, not significant by the *F*-test. Lowercase letters indicate differences in relation to Si and capital letters in relation to water deficit. *n* = 5. Si × WD: interaction.

The C:Si ratio in leaves and stems of plants with -Si was decreased when under WD until the tillering phase (Figures 5A–D) or until the stem’s initial formation (Figures 6A–D) compared to control plants. The Si supply in the seedling production phase only and when supplemented after transplanting decreased the C:Si ratio in the leaves and stems of sugarcane (experiment I and III) and energy cane (experiment II and IV) under the control regime and WD in both periods compared to plants -Si (Figures 5A–D, 6A–D).

Compared to control plants (Figures 5E–H), the C:N ratios in leaves and stems in sugarcane and energy cane and the C:P ratio in sugarcane stems decreased when Si was not applied and the plants were grown under WD regime until the tillering phase (experiment I and II). Under WD until the stem’s initial formation, compared to control plants, the C: N and C: P ratios in leaves and stems were decreased regardless of Si supply in sugarcane (experiment III) (Figures 6E–H) and energy cane (experiment IV) (Figures 6E–L).

The Si supply increased the C:N ratio in leaves and stems of sugarcane (experiment I and III) and energy cane (experiment II and IV) under of both periods of WD compared to -Si (Figures 5E–H, 6G), except for sugarcane leaves in the WD regime until the stem’s initial formation phase (Figure 6A).

The Si supply increased the C:P ratio in sugarcane leaves (Figure 5I) under WD until the tillering phase compared with control plants. However, C:P ratio decreased in sugarcane stems (Figure 5J) and leaves and energy cane stems (Figures 5K,L) under WD until the tillering phase (experiment I and II), and in energy cane leaves and stems under WD until the stem’s initial formation phase (Figures 6I–L).

The N:P ratios in leaves and stems of sugarcane and energy cane increased with the WD regime until the tillering phase, but only in plants -Si (experiment I and II) (Figures 5M–P). However, the N:P ratio increased in both Si supply conditions under WD regime until the stem’s initial formation phase compared to the control regime, except in the sugarcane leaves (experiment III and IV) (Figures 6M–P).

### Relative Water Content and Leaf Water Potential

**FIGURE 7:**
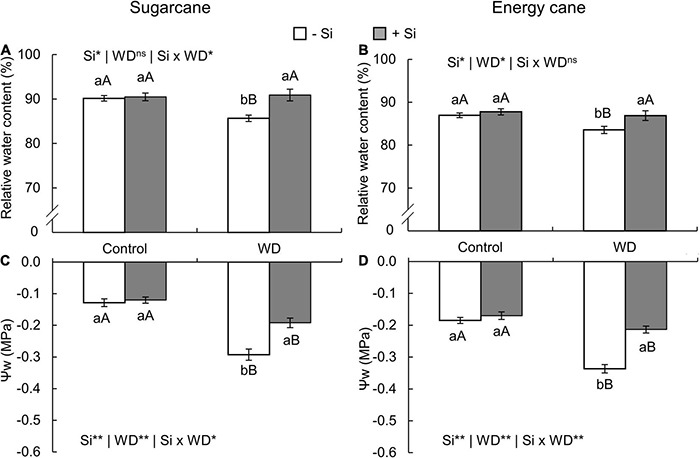
Relative water content and leaf water potential (Ψw) in the sugarcane **(A,C)** and energy cane plants **(B,D)** in -Si and + Si under adequate water regime (control) and in WD for a period of 7 to 60 days after transplantation. ** and * significant with 1 and 5% probability, respectively, and ns, not significant by the *F*-test. Lowercase letters indicate differences in relation to Si and capital letters in relation to water deficit. *n* = 6. Si × WD: interaction.

**FIGURE 8:**
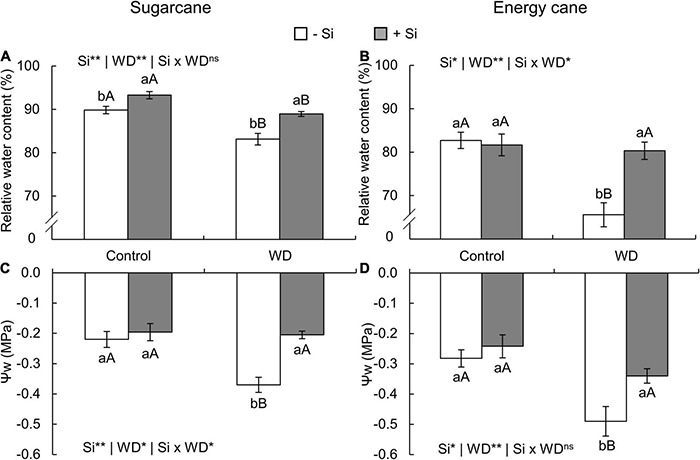
Relative water content and Ψw in sugarcane **(A,C)** and energy cane plants **(B,D)** in -Si and + Si under adequate water regime (control) and in WD for a period of 7 to 160 days after transplantation. ** and * significant with 1 and 5% probability, respectively, and ns, not significant by the *F*-test. Lowercase letters indicate differences in relation to Si and capital letters in relation to water deficit. *n* = 5. Si × WD: interaction.

In both species, the relative water content in leaf tissue decreased in plants with -Si and under WD until the tillering phase (experiment I and II) (Figures 7A,B) and until the stem’s initial formation (experiment III and IV) (Figures 8A,B) when compared to control plants. On the other hand, Si supply increased the water content in the leaf tissue of sugarcane (Figures 7A, 8A) and energy cane (Figures 7B, 8B) under control regime and WD compared to -Si.

The Ψw decreased in the two species under WD during both periods, especially in -Si. However, Si supply kept the leaf water potential higher, when compared to -Si and under WD in sugarcane (experiment I and III) (Figures 7C, 8C) and energy cane (experiment II and IV) (Figures 7D, 8D).

### Carbon, Nitrogen, and Phosphorus Use Efficiencies and Dry Matter Production

**FIGURE 9:**
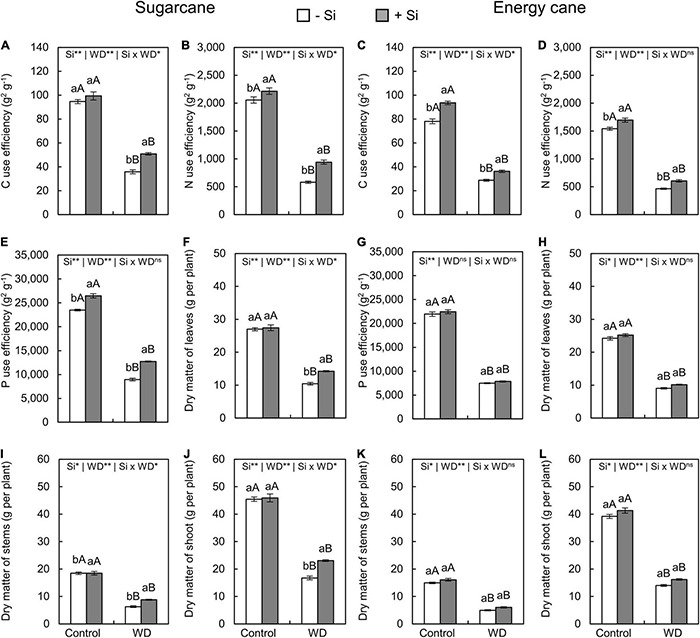
Carbon, N, and P use efficiencies, dry matter of leaves, stems, and shoot of sugarcane **(A,B,E,F,I,J)** and energy cane **(C,D,G,H,K,L)** in -Si and + Si under adequate water regime (control) and in WD for a period of 7 to 60 days after transplantation. ** and * significant with 1 and 5% probability, respectively, and ns, not significant by the *F*-test. Lowercase letters indicate differences in relation to Si and capital letters in relation to water deficit. *n* = 6. Si × WD: interaction.

**FIGURE 10:**
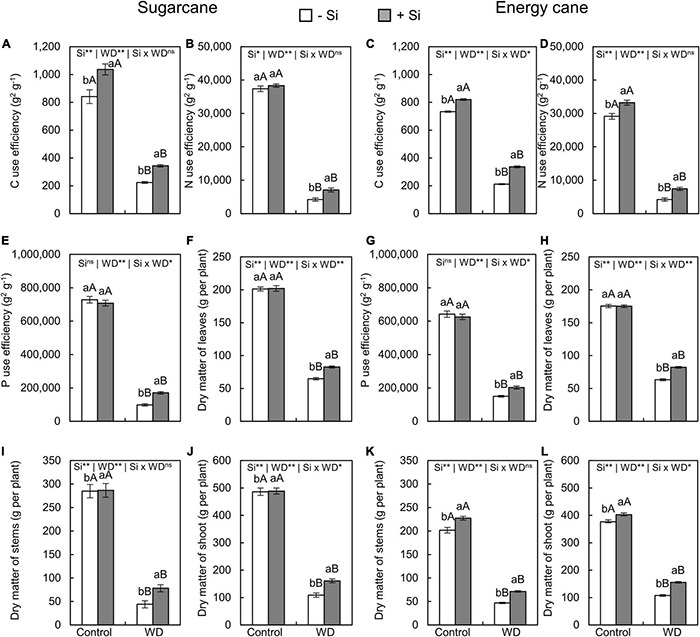
Carbon, N, and P use efficiencies, dry matter of leaves, stems, and shoot of sugarcane **(A,B,E,F,I,J)** and energy cane **(C,D,G,H,K,L)** in -Si and + Si under adequate water regime (control) and in WD for a period of 7 to 160 days after transplantation. ** and * significant with 1 and 5% probability, respectively, and ns, not significant by the *F*-test. Lowercase letters indicate differences in relation to Si and capital letters in relation to water deficit. *n* = 5. Si × WD: interaction.

The use efficiencies of C, N, and P decreased in plants of both species subjected to the two periods of WD compared to control plants (Figures 9, 10A–G). However, the Si supply, compared to -Si, increased use efficiencies of C and N in sugarcane and energy cane grown under both periods of WD (experiment I–IV) (Figures 9A–D, 10A–D).

The Si supply increased P use efficiency only in sugarcane under WD until the tillering phase (experiment I) and in both species under WD until the stem’s initial formation phase (experiments III and IV) compared to -Si plants (Figures 9, 10E,G).

The dry matter of leaves (Figures 9, 10F,H), stems (Figures 9, 10I,K), and shoots (Figures 9, 10J,L) decreased when plants were grown under WD until the tillering and stem’s initial formation phases in both species, regardless of Si supply.

The Si supply during seedling formation only, compared to -Si, provided decreasing dry matter losses in sugarcane under WD until the tillering phase (Figures 9F–L), with 37%, 40%, and 38% greater leaf, stem, and shoot dry matter, respectively (experiment I) (Figures 9F,I,J). Nevertheless, when plants were also enriched with Si by application after transplanting, compared to -Si, an increase of 28%, 78%, and 48% was observed in sugarcane (experiment III), while 30%, 52%, and 45% was observed in energy cane (experiment IV) of leaf, stem, and shoot dry matter, respectively, in plants under WD until the stem’s initial formation phase (Figures 10F–L).

## Discussion

The Si supply interferes with the stoichiometric ratio of Si:C:N:P, increasing the use efficiencies of C, N, and P with repercussions on the dry matter production of sugarcane and energy cane grown under severe WD. These effects are influenced by the time the applications are made and the WD condition. Can be obtained with Si supply exclusively in the seedling formation phase in plants under severe WD up to 60 days after transplanting. However, extend up to 160 days when there is a Si supply with fertigation and foliar spraying after transplanting. These effects vary with the species as it is more evident in sugarcane than energy cane.

The Si benefit on plants depends on its uptake capacity and on the growing conditions or application times. Plants grown in soils with low Si content, such as the Entisols (Quartzipsamments) (Si = 1.0 mg dm^–3^) used in this research, are more affected (Schaller et al., 2020). Plants of the Poaceae family absorb more Si because they have proteins in the root tissue membranes specialized in active Si uptake and transport (Mitani-Ueno and Ma, 2021). Although there are no previous reports on the capacity of first cycle energy cane (plant cane) to absorb Si, the results obtained in this research show that the increase in the Si content of leaves and stems was similar to that obtained in sugarcane. Furthermore, it indicates for the first time that energy cane can also be classified as a Si accumulator. This is due to our observation that the Si application resulted in Si increase in the plant compared to the control.

The high Si uptake is also due to the form used for its application. It has been shown that fertigation associated with foliar spraying is efficient in providing relevant increases in Si content on both species’ leaves and stems. This effect can be attributed to the quality of the applied solution that possibly had low polymerization rates due to the adequate Si concentration used (2.5 mmol L^–1^). Hence, keeping the element in the monomeric form (H_4_SiO_4_) (Birchall, 1995). Furthermore, this form of Si application allowed the use of a soluble source stabilized with sorbitol, a polyol that helps maintain the Si monomeric forms by forming organic complexes (Kubicki and Heaney, 2003). However, more research is needed to confirm this preliminary indication.

According to literature, sorbitol also decreases the deliquescence point of the droplet on the leaf surface (Babiker and Duncan, 1974), delaying its drying and contributing to the element’s uptake by the leaf surface, especially during the cuticle penetration phase (Fernández and Brown, 2013). Another important aspect used to maintain the Si monomeric form was acidification at the time of application, lowering the solution’s pH value (5.5 ± 0.2). In acidic solutions containing Si, the reaction shifts to the right (H_2_SiO_4_^–2^ → H_3_SiO_4_^–1^ → H_4_SiO_4_), and monomeric species of the element predominate (Kudryavtsev and Figovsky, 2016). Together, these factors improved the quality of the Si solution applied to the plants.

The adequate handling of the Si solution supplied to the plant favored its uptake by the leaf and root organs, increasing the Si contents in both species. The Si application that was exclusive to the seedling formation phase increased the Si content in both species’ leaves and stems compared to Si-untreated plants. However, by complementing with Si fertigation after transplanting, the effect on the increase of leaf and stem contents was even more expressive, especially in sugarcane. Thus, both forms of supply can be considered for adequate plant nutrition with Si since fertigation associated with foliar spraying is already a technique used in commercial sugarcane seedling production nurseries for nutrient application (Landell et al., 2012). The complementation after transplanting also has applicability because it can be performed during localized irrigations that are common after transplanting the seedlings due to low rainfall at the time of planting (ANA, 2021).

The high Si uptake in plant tissue predominantly decreased the C contents of leaves and stems in plants of both species grown under the control and in both periods of WD. The incorporation of Si into plant tissues can partially replace C in cell wall organic compounds (Neu et al., 2017). This is because Si is immobilized in cell walls as phytoliths, which are structural materials consisting of a combination of biomolecules (Schoelynck et al., 2010). This substitution is of biological interest because the energy cost (NADPH and ATP) for incorporating Si into structural compounds, compared to organic compounds, is 50 times lower due to the high intrinsic permeability in lipid bilayers (Hao et al., 2020). This indicates an induced competitive advantage by Si as plants spend less energy forming these high-cost metabolic compounds (Schoelynck et al., 2010; Schaller et al., 2012). Therefore, plants can direct energy to optimize the defense metabolism against WD.

The Si-induced decline in leaf tissue C concentrations has also been reported in wheat (Neu et al., 2017) and *Phragmites australis* (Schaller et al., 2012) without WD and in sorghum grown under salinity stress (Hurtado et al., 2020). Given the results in this study, it is evident that the Si-induced lower C content also occurs in the leaves and stems of sugarcane and energy cane under adequate water regimes and severe WD until the tillering phase and in the stem’s initial formation.

The severe WD imposed on sugarcane and energy cane for up to 60 and 160 days after transplanting increased N content in leaves and stems, especially in plants that did not receive Si supply. The high N content suggests a possible plant strategy to attenuate WD by increasing the content of N-containing osmoprotective compounds, such as proline. Proline promotes cellular osmotic adjustment to maintain the tissue water status (Szabados and Savouré, 2010). Different results were obtained in *Stylosanthes capitata* Vogel (Viciedo et al., 2021) and *Panicum maximum* Jacq (Viciedo et al., 2019) grown under a WD induced by the total absence of irrigation for 30 days. Thus, this survey’s results indicate that when plants are grown with the continuous supply of a lower amount of water (30% WRC) for intermediate and long periods, N uptake is not limited, and plants can acquire osmotic adjustment mechanisms as a drought tolerance strategy.

However, the Si supply decreased the N contents in leaves and stems of sugarcane and energy cane grown under WD until the tillering and stem’s initial formation phases, except in sugarcane leaves under WD in the period up to 160 days after transplanting. This effect can be justified by a dilution of the nutrient content, given an increase in dry matter production. The N accumulation in shoots of sugarcane under WD until 60 days after transplanting were.6 and.5 g per plant in + Si and -Si, respectively. In energy cane, they were.45 and.42 g per plant at + Si and -Si, respectively. Under WD, up to 160 days after transplanting, the N accumulations in the sugarcane shoot were 3.7 and 2.7 g per plant in + Si and -Si, respectively. In energy cane, they were 3.3 and 2.8 in + Si and -Si, respectively.

Another effect that may account for the decrease in N contents in plants that received Si supply suggests that the element exerted a beneficial effect on balancing the plants’ water status. Previous reports indicate maintenance of water status in plants that absorb large amounts of Si due to the deposition of cellulose-associated silica in the leaf epidermis and below the cuticle (Mitani-Ueno and Ma, 2021). This deposition acts as a barrier to water loss and decreases transpiration. This effect was confirmed by the higher water content in leaf tissues benefiting from the Si-induced adjustment in water potential, as observed in plants under + Si and intermediate and long-term WD. The Si-mediated high relative water content decreased oxidative stress and prevented damage from excessive ROS accumulation (Yin et al., 2005). The Si supply through the roots in wheat without WD also induced a decline in N contents (Murozuka et al., 2014).

Growing plants without Si supply and under severe WD until the tillering phase increased P contents only in the sugarcane stems. However, when plants were grown under WD until the stem’s initial formation phase, P contents was increased in leaves and stems of both species. The increase in P contents can be justified by the decrease in high-energy-cost biological processes such as the enzymatic activity of different biochemical pathways involved in amino acid formation and protein synthesis. Suspending water supply in *P. maximum* Jacq (Viciedo et al., 2019) *and S. capitata* Vogel (Viciedo et al., 2021) plants for 30 days limited P uptake with a decrease in leaf contents. Therefore, this survey’s results demonstrate that WD induced by supply smaller amounts of water (30% of WRC) that was applied daily for 7 to 60 or 160 days after transplanting does not limit P uptake by sugarcane and energy cane.

The Si supply increased P contents in energy cane under both periods of WD. The increase in P content and plant dry matter is strong evidence of increased nutrient uptake and transport to the shoot. This fact can be observed when analyzing the values of P accumulation in shoots of sugarcane under WD for up to 60 days after transplanting (i.e., 42 and 32 mg per plant in + Si and -Si, respectively). In energy cane, they were 35 and 25 mg per plant at +Si and -Si, respectively. A similar fact was observed in the WD condition until 160 days after transplanting, where the P accumulation in shoots of sugarcane were 157 and 120 mg per plant in + Si and -Si, respectively. In energy cane, they were 121 and 77 mg per plant at + Si and -Si, respectively.

Similar results have been reported for sugarcane grown under WD with Si supply via root only (Teixeira et al., 2020b) and in rice (Ma and Takahashi, 1990) and sugarcane (Frazão et al., 2020) grown without deficit. However, in sugarcane, the Si supply decreased the P content in the stems of plants under the WD regime until the stem’s initial formation phase, indicating that the Si supply and stress level interfere in P metabolism, with consequent effects in its stoichiometry and efficiency of use.

The changes in the nutrient contents interfered in their proportions in both species’ leaves and stems. The severe WD condition without Si supply decreased the C:Si ratio in leaves and stems of sugarcane and energy cane under in both periods of WD duration. Plants that received Si supply showed no difference regarding the water regime, presenting low C:Si ratios in both species’ leaves and stems. The lower C:Si ratio occurred by decreasing C content and increasing Si content.

The C:N ratio in leaves and stems of sugarcane and energy cane decreased in plants grown without Si application and WD during both periods. The C:N ratio decrease is mainly due to the increased N contents observed in water-deficient plants for both duration periods. However, the Si supply in the seedling formation phase provided an increase in the C:N ratio in the leaves and stems of sugarcane and energy cane even under severe WD until the tillering phase and was indeed similar to that of unstressed plants in the leaves of both species. Under WD for a longer period and until the stem’s initial formation phase, there was an increase in the C:N ratio only in the energy cane leaves and the sugarcane stems. It demonstrates that the Si effect on the C:N ratio varies with the WD duration, the evaluated organ, and the species.

The decreasing Si effect on C content that is associated with the increasing P content in energy cane grown under the two WD duration periods decreased the C:P ratio in leaves and stems. Nevertheless, in sugarcane, the C:P ratio did not follow the same pattern, decreasing only in the stems in plants grown under WD until the tillering phase. The lower C:P ratios in leaves and stems indicate that Si contributes to maintaining the balance between C and P even in water-deficient plants. Thus, the Si uptake increase, especially in stressed plants, contributes to maintaining homeostasis and balancing the nutrient ratios in leaves and stem tissue, increasing the amount of C-rich structural components with metabolic function to the detriment of structural function.

Sugarcane and energy cane under WD and no Si supply had lower C use efficiency and biomass production. This can be explained by a decrease in nutrient redistribution, which is common in plants under WD (Brouder and Volenec, 2008). In turn, Si that is exclusively applied through fertigation and foliar spraying in the seedling formation phase with application after transplanting increased the C use efficiency of sugarcane and energy cane under severe WD. It can be attributed to the greater Si incorporation to plant tissues, with its use being more in photosynthesis than in structural compounds (Schaller et al., 2012) as C is replaced by Si (Hao et al., 2020).

Nitrogen and P use efficiencies were also decreased in sugarcane and energy cane that did not receive Si supply when grown under both periods of WD. However, Si-induced optimization of C metabolism improved C skeleton production and the structural nutrient metabolism (e.g., N and P that constitute vital organic compounds for the plant) (Prado, 2021), partially decreasing losses caused by the WD. The improvement in N use efficiency by Si supply may result from changes in primary metabolism, stimulating the translocation of amino acids from the source to the absorbing tissues (Detmann et al., 2013).

The increased C and N use efficiencies indicate decreased energy cost in the Si incorporation by substitution of organic compounds, reflecting on the increased P use efficiency. This increase in N and P use efficiencies due to the Si effect has already been reported in unstressed winter wheat (Neu et al., 2017) and in pre-sprouted sugarcane seedlings under WD for up to 30 days (Teixeira et al., 2020b).

The beneficial effects of Si in alleviating the damage caused by WD in sugarcane through adjustment of physiological and biochemical parameters have been previously described (Bezerra et al., 2019; Teixeira et al., 2020a; Verma et al., 2021). However, there are no reports to relate the gains in plant dry matter production to Si:C:N:P stoichiometry changes. It indicates that the Si effect on increasing nutritional homeostasis and use efficiencies of C, N, and P was limited to sugarcane under a maximum WD of 30 days after transplanting (Teixeira et al., 2020b) and to sugarcane without water stress (Frazão et al., 2020). Thus, the results evidenced here indicate for the first time that the Si effects on Si:C:N:P stoichiometric ratios and on C, N, and P use efficiencies have a very relevant beneficial potential in alleviating severe WD for intermediate (up to 60 days) and long (up to 160 days) periods. Therefore, these effects should be considered to understand better how Si contributes to WD alleviation in sugarcane and energy cane grown under this condition until the tillering or stem’s initial formation phases.

A beneficial effect of Si was observed in increasing P use efficiency in energy cane that received Si application only during the seedling production phase. This fact may justify the lack of the element’s effect on the dry matter increment of leaves, stems, and shoots of energy cane cultivated under WD until the tillering phase. The absence of beneficial mitigating Si effect observed in energy cane that was grown under severe WD from 7 to 60 days after transplanting can be attributed to lower Si uptake. This is because Si was applied only during seedling formation, and because about 1/3 of the leaf biomass was removed just before transplanting the plant into the soil. Also, there was no Si effect on increasing the P use efficiency in this species, and although it did affect the other nutrients, this contribution was insufficient to improve the energy cane biomass production. Thus, the P use efficiency seems to be an important mechanism that Si modifies to alleviate the WD in the earliest phase of the crop, i.e., the plant’s tillering.

These results allow us to accept the hypothesis that the WD damage to sugarcane and energy cane dry matter production is due to an imbalance in nutritional homeostasis that decreases C, N, and P use efficiencies. However, the results also show that Si supply can reverse these effects. To a greater degree, this effect was evident when the plants were supplied with more applications of the beneficial element, with fifteen applications in the seedling production phase and with more applications after transplanting to the field (experiments III and IV), benefiting both species under study. On the other hand, when the plant received fewer applications of Si, restricted to only fifteen applications in the seedling formation phase (experiments I and II), benefiting only the sugarcane.

This study proposes for the first time that the Si supply optimization alleviates the WD damage in sugarcane and energy cane for an intermediate and long period. Although these stages are more sensitive to water stress, optimizing the element supply corresponds to a major advance in these crops’ planting systems. For the first time, the potential of Si in solution (fertigation and foliar spraying) applied during seedling formation and initial plant growth at relatively low doses (<20 kg ha^–1^ of Si) is indicated without any risks to the environment but is sufficient for better sustainable use of water (more biomass with less water) in these crops’ critical establishment period.

## Conclusion

The damage caused by WD in plants is due to the imbalance of nutritional homeostasis that decreases the C, N, and P use efficiencies and, consequently, the biomass accumulation of sugarcane and energy cane. However, depending on the and the period of WD, the Si supply through fertigation and foliar spraying can reverse this situation by increasing the C, N, and P use efficiencies.

The research proposes the Si supply in the seedling production phase to alleviate the intermediate severe WD. It means up to 60 days after transplanting only for the sugarcane crop, but if the beneficial element is complemented after plant transplanting, it is possible to alleviate this water stress extending up to 160 days after transplanting for the two species studied.

This research shows that optimizing the Si application is a new strategy to improve plant tolerance to drought stress in sugarcane and energy cane. However, because of the changes in nutrient contents in plant tissue, field trials would be needed to tailor the strategy to be applied in the field.

## Data Availability Statement

The raw data supporting the conclusions of this article will be made available by the authors, without undue reservation.

## Author Contributions

GT did the conceptualization, carried out the data curation and formal analysis, investigated the data, performed the methodology, wrote the original draft of the manuscript, and wrote, reviewed, and edited the manuscript. RM did the conceptualization, carried out the funding acquisition, project administration, resources, performed the methodology, supervised the data, and wrote, reviewed, and edited the manuscript. AR carried out the formal analysis, investigated the data, performed the methodology, and wrote the original draft of the manuscript. MC carried out the resources and funding acquisition and wrote the original draft of the manuscript. All authors contributed to the article and approved the submitted version.

## Conflict of Interest

The authors declare that the research was conducted in the absence of any commercial or financial relationships that could be construed as a potential conflict of interest.

## Publisher’s Note

All claims expressed in this article are solely those of the authors and do not necessarily represent those of their affiliated organizations, or those of the publisher, the editors and the reviewers. Any product that may be evaluated in this article, or claim that may be made by its manufacturer, is not guaranteed or endorsed by the publisher.
